# Supplementary material for: Optimization of the Freezing-Thawing Method for Extracting Phycobiliproteins from Arthrospira sp
Source: Molecules. 2020 Aug 26;25(17):3894. doi: 10.3390/molecules25173894 (PMC7503228; doi:10.3390/molecules25173894)
Supplement: Supplementary file 1 [file molecules-25-03894-s001.pdf]

Article

Optimization of the Freezing-Thawing Method for Extracting Phycobiliproteins from *Arthrospira* sp.

Hui Teng Tan [1], Nicholas M. H. Khong [2], Yam Sim Khaw [1], Siti Aqlima Ahmad [3] and Fatimah M. Yusoff [4,5,*]

[1] Marine Biotechnology Laboratory, Institute of Bioscience, Universiti Putra Malaysia, 43400 Serdang, Selangor Darul Ehsan, Malaysia; huiteng.tan28@gmail.com (H.T.T.); yskhaw@gmail.com (Y.S.K.)
[2] School of Pharmacy, Monash University Malaysia, Jalan Lagoon Selatan, 47500 Bandar Sunway, Selangor Darul Ehsan, Malaysia; nicholas.khong@monash.edu
[3] Department of Biochemistry, Faculty of Biotechnology and Biomolecular Sciences, Universiti Putra Malaysia, 43400 Serdang, Selangor Darul Ehsan, Malaysia; aqlima@upm.edu.my
[4] Department of Aquaculture, Faculty of Agriculture, Universiti Putra Malaysia, 43400 Serdang, Selangor Darul Ehsan, Malaysia
[5] International Institute of Aquaculture and Aquatic Sciences, Universiti Putra Malaysia, 43400 Serdang, Selangor Darul Ehsan, Malaysia
* Correspondence: fatimamy@upm.edu.my; Tel.: +603-89408311

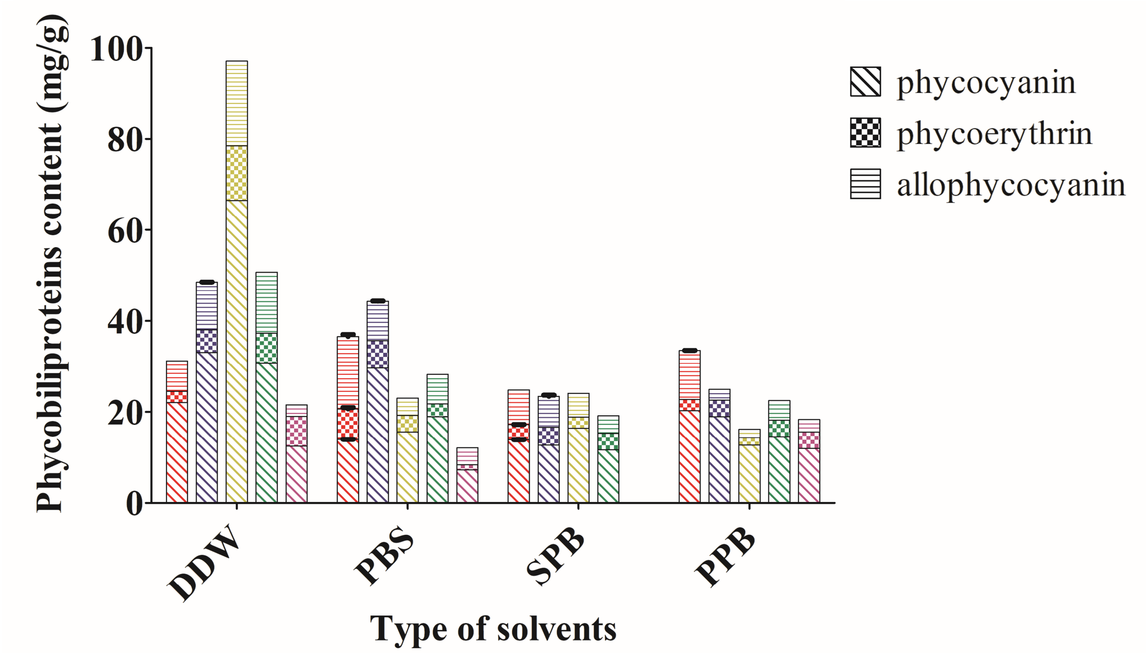

Figure S1. Total phycobiliproteins content extracted from *Arthrospira* sp. (UPMC-A0087) using different solvents with varying pH values.

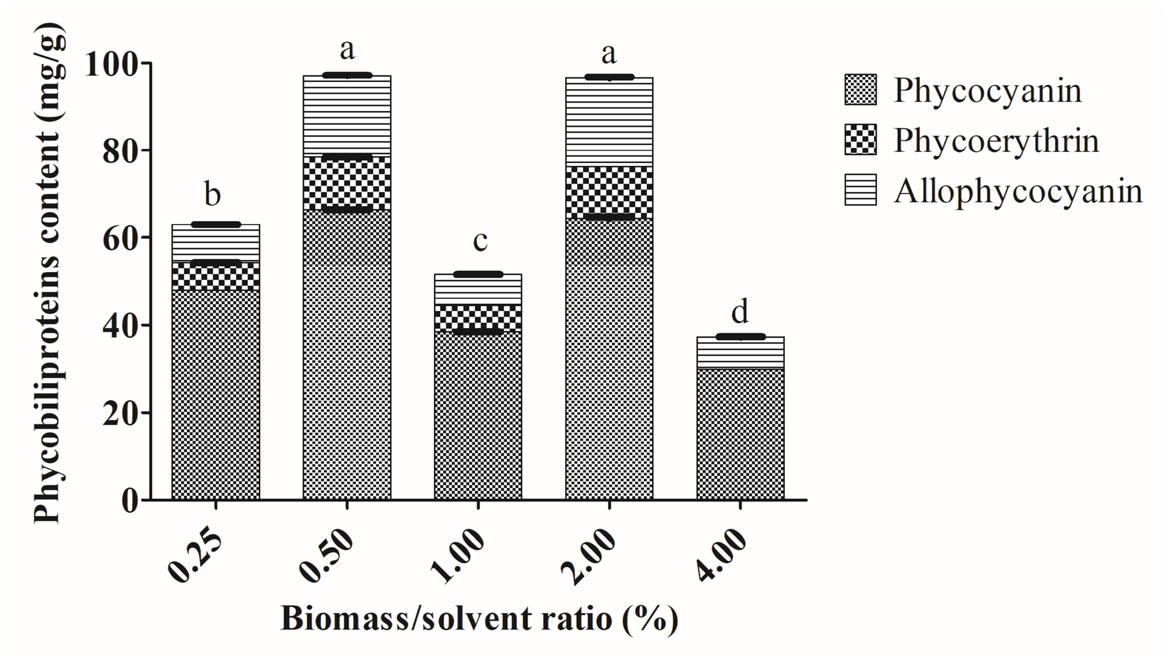

Figure S2. Extractions of phycobiliproteins content from *Arthrospira* sp. (UPMC-A0087) using different biomass solvent ratios.

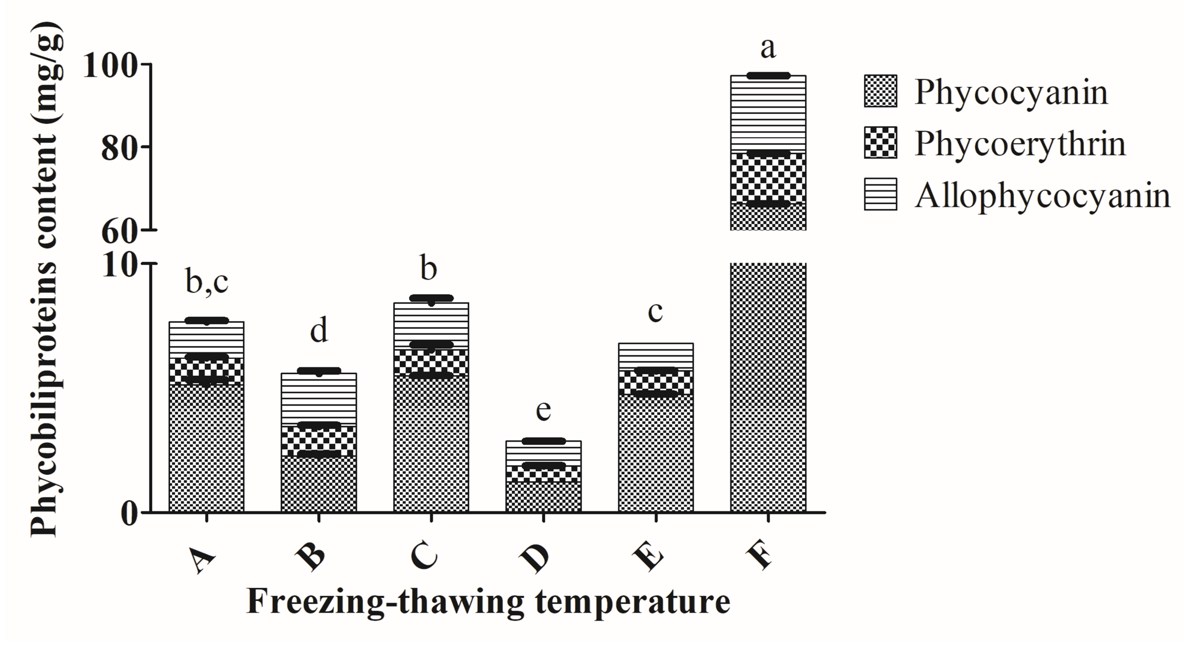

Figure S3. Extractions of phycobiliproteins content of *Arthrospira* sp. (UPMC-A0087) using different freezing–thawing temperatures.

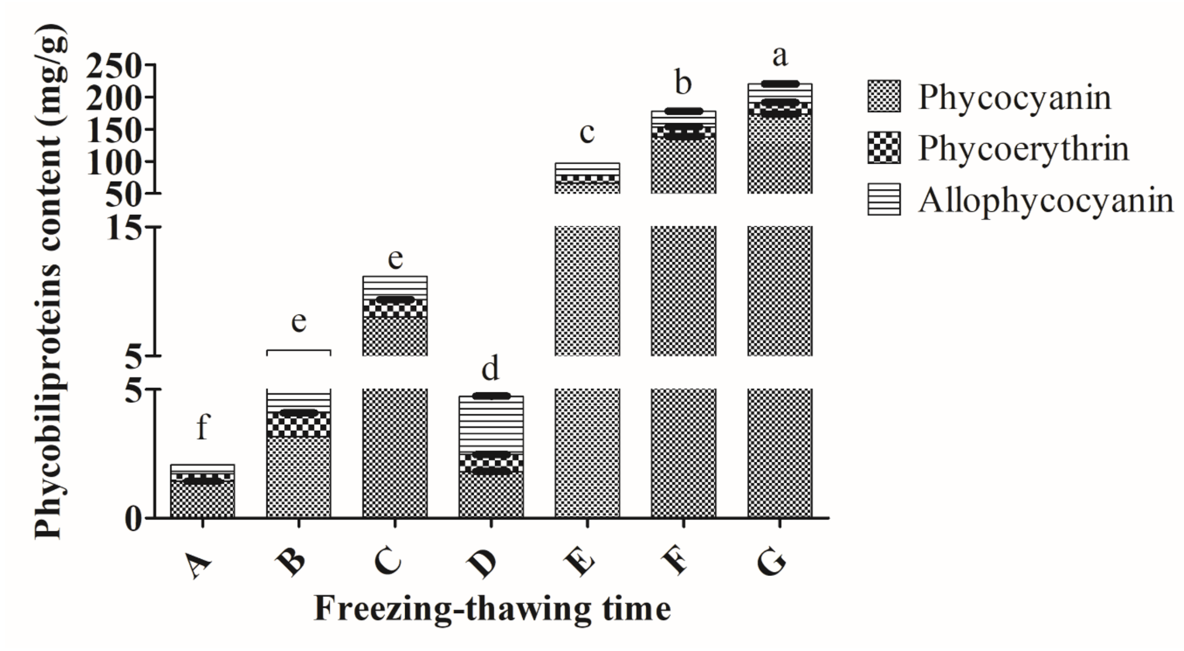

Figure S4. Extractions of phycobiliproteins from *Arthrospira* sp. (UPMC-A0087) using different freezing–thawing times.

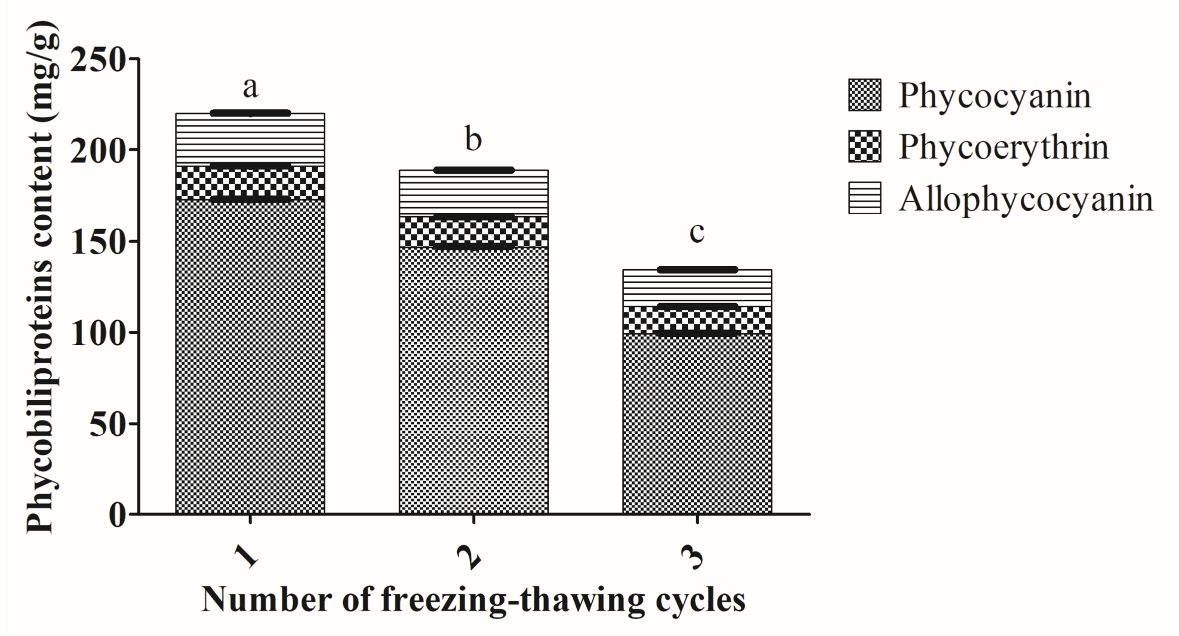

Figure S5. Extractions of phycobiliproteins content from *Arthrospira* sp. (UPMC-A0087) using different number of freezing–thawing cycles.

Table S1. Purity of extracted phycobiliproteins using different solvents with diverse pH. (A) Phycocyanin; (B) Phycoerythrin; (C) Allophycocyanin.

	**A**	
**Solvent**	**pH**	**Phycocyanin purity (A620/A280)**
	6	0.82 ± 0.14
	6.5	0.72
Water	7	0.85 ± 0.01
	7.5	0.44 ± 0.03
	8	0.31 ± 0.01
Phosphate saline buffer	6	0.56 ± 0.01

	6.5	0.74
	7	0.26
	7.5	0.40
	8	0.30
Sodium phosphate buffer	6	0.20
	6.5	0.30
	7	0.35
	7.5	0.20
Potassium phosphate buffer	6	0.31
	6.5	0.65
	7	0.33 ± 0.03
	7.5	0.33 ± 0.03
	8	0.34 ± 0.01

B

**Solvent**	**pH**	**Phycoerythrin purity (A562/A280)**
Water	6	0.46 ± 0.08
	6.5	0.27
	7	0.40
	7.5	0.30 ± 0.04
	8	0.20 ± 0.01
Phosphate saline buffer	6	0.40 ± 0.01
	6.5	0.49
	7	0.17
	7.5	0.25
	8	0.19
Sodium phosphate buffer	6	0.14
	6.5	0.20
	7	0.21
	7.5	0.14
Potassium phosphate buffer	6	0.17
	6.5	0.39
	7	0.19
	7.5	0.23
	8	0.22 ± 0.01

C

**Solvent**	**pH**	**Allophycocyanin purity (A652/A280)**
Water	6	0.36 ± 0.06
	6.5	0.33
	7	0.44 ± 0.01
	7.5	0.23 ± 0.03
	8	0.11 ± 0.01
Phosphate saline buffer	6	0.35 ± 0.01
	6.5	0.32
	7	0.1
	7.5	0.15
	8	0.11
Sodium phosphate buffer	6	0.12
	6.5	0.13
	7	0.13
	7.5	0.09
Potassium phosphate buffer	6	0.13
	6.5	0.20
	7	0.11
	7.5	0.15
	8	0.13 ± 0.01

Table S2. Purity of extracted phycobiliproteins using different biomass solvent ratio.

Biomass solvent ratio	Phycocyanin purity (A620/A280)	Phycoerythrin purity (A562/A280)	Allophycocyanin purity (A652/A280)
0.25	0.62	0.34	0.22
0.50	0.85 ± 0.01	0.40	0.44 ± 0.11
1.00	1.06	0.60	0.38
2.00	0.15 ± 0.02	0.07 ± 0.01	0.06 ± 0.01
4.00	1.94	1.09	0.77

Table S3. Purity of extracted phycobiliproteins using different freezing-thawing temperature.

Freezing-thawing temperature	Phycocyanin purity (A620/A280)	Phycoerythrin purity (A562/A280)	Allophycocyanin purity (A652/A280)
Freeze 0 ^{0}C; Thaw 4 ^{0}C	0.23 ± 0.01	0.16	0.10
Freeze 0 ^{0}C; Thaw 25 ^{0}C	0.11	0.11	0.07
Freeze -30 ^{0}C; Thaw 4 ^{0}C	0.29 ± 0.06	0.20	0.14
Freeze -30 ^{0}C; Thaw 25 ^{0}C	0.09	0.09	0.06
Freeze -80 ^{0}C; Thaw 4 ^{0}C	0.31	0.20	0.12
Freeze -80 ^{0}C; Thaw 25 ^{0}C	0.85 ± 0.01	0.40	0.44 ± 0.11

Table S4. Purity of extracted phycobiliproteins using different freezing-thawing time.

Freezing-thawing time	Phycocyanin purity (A620/A280)	Phycoerythrin purity (A562/A280)	Allophycocyanin purity (A652/A280)
Freeze 0.5 h; Thaw 1 h	0.22	0.14	0.08
Freeze 0.5 h; Thaw 1.5 h	0.19	0.14	0.09
Freeze 0.5 h; Thaw 2 h	0.39	0.23	0.15
Freeze 1 h; Thaw 2 h	0.17	0.20	0.15
Freeze 2 h; Thaw 2 h	0.85 ± 0.01	0.40	0.44 ± 0.11
Freeze 2 h; Thaw 12 h	0.84	0.30	0.23
Freeze 2 h; Thaw 24 h	1.95	0.50	0.96

Table S5. Purity of extracted phycobiliproteins using different number of freezing-thawing cycles.

Freezing-thawing cycle	Phycocyanin purity (A620/A280)	Phycoerythrin purity (A562/A280)	Allophycocyanin purity (A652/A280)
1	1.95	0.50	0.96
2	0.61 ± 0	0.30	0.56
3	0.50 ± 0	0.15	0.24

Table S6. Phycobiliproteins content extracted by optimized condition after stored 24 hours.

**Phycobiliproteins**	**Amount of phycobiliproteins content (mg/g)**		
	0 hour	***24 hours***	***Loss of content (%)***
Phycocyanin	172.84 ± 0.37	164.55 ± 0.17	4.80
Phycoerythrin	18.14 ± 0.38	15.29 ± 0.49	15.71
Allophycocyanin	28.89 ± 0.50	25.77 ± 0.37	10.80
Total phycobiliproteins	219.87 ± 0.68	205.60 ± 0.94	6.49

*Values represent mean (± standard deviation) of 3 replicates. *Values reported in mg/g.

Table S7. Phycobiliproteins extraction conditions that used in previous studies.

Extraction solvent	Biomass-solvent ratio (%)	Temperature ⁰C		Time (min)		Freezing-thawing cycle	Yield (mg/g)	Purity	Reference
		Freezing	Thawing	Freezing	Thawing				
Phosphate buffer (pH 6.8)	16.67	-40	RT	240	60	4	*Arthrospira platensis*: 73.73 (PC)	0.66	[40]
Phosphate buffer (pH 7)	n.a	-21	4	240	240	n.a	*Spirulina* sp. : 86.3 ± 1.1 (PC)	1.34 ± 0.05	[33]
Sodium phosphate buffer (pH 7)	n.a	-20	4	180	5	3	*Spirulina platensis*: 146 (PC)	0.87	[36]
Sodium phosphate buffer (pH 7.5) supplemented with sodium chloride	100	-20	4	n.a	n.a	n.a	*Anabaena* sp.: 128 ± 0.13 (TPB)	0.87	[24]
Sodium phosphate buffer (pH 7)	50	n.a	n.a	n.a	n.a	2	*Spirulina platensis*: 0.76 (PC)	n.a	[22]
Tris-Cl buffer (pH 8.1)	20	-25	4	n.a	n.a	4	*Oscillatoria quadripunctulata*: 137.15 (PC)	0.85	[34]
Sodium phosphate buffer (pH 7.0)	2	-20	RT	n.a	n.a	3	*Nostoc commune* TUBT05: 29.66 ± 0.52 (PC)	0.62 ± 0.02	[50]
Tris-HCl buffer (pH 8)	2	-20	RT	n.a	n.a	18	*Oscillatoria okeni* TISTR8549: 39.93 ± 0.9 (PC)	1.65 ± 0.02	[50]
Sodium phosphate buffer (pH 7) containing 0.0025 mg/mL lysozyme	3	-20	4	300	At least 240	n.a	*Euhalothece* sp.: 75 (PC)	2.5	[37][a]

[a]Incubation for 2 hours at 37 °C prior to thawing process

*n.a, not available; TPB, total phycobiliprotein, PC, phycocyanin, RT, room temperature
